# Short- and Long-Term Retentivity of Knowledge by Various Teaching Methods in Medical Education and Perception of Students Towards Them: A Comparative Study in a Medical University Hospital of Northern India

**DOI:** 10.7759/cureus.48043

**Published:** 2023-10-31

**Authors:** Shubhajeet Roy, Narendra Kumar, Vaishali Singh, Sarvesh Singh, Rahul Kumar, Jay Tewari, Darshit Samaiya, Amod K Sachan

**Affiliations:** 1 Faculty of Medical Sciences, King George's Medical University, Lucknow, IND; 2 Pharmacology and Therapeutics, King George's Medical University, Lucknow, IND; 3 Pharmacology and Therapeutics, King George’s Medical University, Lucknow, IND; 4 Faculty of Medical Sciences, King George’s Medical University, Lucknow, IND

**Keywords:** self-learning, app based learning, black-board learning, powerpoint presentation, medical education, teaching methods

## Abstract

Background: To develop doctors with appropriate knowledge of health and diseases, reasonable medical abilities, and a positive attitude toward patients and their families, it is important to reexamine the methods used to educate and train medical school students. To establish which is best for both medical students and professors, the various teaching and learning methodologies must be compared and analyzed. This study attempts to determine the preferred medical education techniques among medical students as well as the caliber of the classes they attend.

Methods: This is a before-and-after study conducted among 480 first- (240) and second-year (240) undergraduate students. Students were divided into three groups. Each group was assigned a teacher who was responsible for teaching four short topics according to the common understanding and knowledge level of both year students in four different ways: traditional blackboard method, offline PowerPoint presentation, online PowerPoint presentation, and online annotative. Application-based learning and self-learning were the other two teaching methods conducted in a monitored environment. An MCQ-based pre- and post-test were taken to assess the improvement, and a feedback form was filled out by each student to assess their perception. To assess long-term retention, a surprise follow-up test was conducted after 15 days.

Results: For all the teaching methods except for traditional blackboard and online presentation, there was a significant improvement in the post-test scores as compared to the pre-test scores (p<0.05). Retentivity was more remarkable in online application-based and self-learning methods. 77.2% of the study participants preferred offline presentation as the mode of teaching.

Conclusion: Retention was found to be highest in self-directed and application-based learning. So, students should be encouraged and motivated for self-study after every lecture, whatever the teaching method used by teachers.

## Introduction

India’s educational institutions (schools, colleges, and universities) only use traditional teaching techniques at this time, which include giving in-person lectures in a classroom [1]. Historically, the majority of medical education has been taught using didactic teaching methods, with reading assignments before and after class [2]. Despite the widespread adoption of blended learning, many medical institutions, including the governing body National Medical Commission (NMC), still prioritize training students using antiquated techniques. However, the COVID-19 epidemic has sped up the trend in medical education that prefers active learning techniques over traditional lectures [3]. 

Infrastructure, patient exposure, faculty subject matter expertise and knowledge, exposure to teaching-learning methodologies, and training are all factors that affect the quality of medical education. These factors are in addition to the curriculum [4]. To help medical students learn more effectively and quickly, a variety of teaching techniques have been used in India to provide medical education, including problem-based learning, didactic lectures, videotapes, seminars, role plays, case studies, and tutorials. For medical education to advance, more focus should be placed on both instructional strategies and technical developments [5].

In many higher education institutions, particularly in medical education, absenteeism is a recurring problem. Absenteeism was substantially correlated with a lack of enthusiasm for the subject, subpar teaching techniques and strategies, illness, excessive social mobilization, a bad rapport with demonstrators, and easy access to online content [5]. Over the past 10 years, a paradigm shift from a teacher-centric to a student-centric approach has taken place to address these issues with the delivery of medical education [4]. It is essential to improve the medical education system, particularly through the implementation of cutting-edge teaching-learning techniques, to encourage learning through student involvement, curiosity, and active participation. To raise the quality of medical education, it is also critical to evaluate students’ perceptions of teaching-learning techniques.

To develop doctors with appropriate knowledge of health and diseases, reasonable medical abilities, and a positive attitude toward patients and their families, it is more important than ever to reexamine the methods used to educate and train medical school students [5]. To establish which is best for medical students and professors alike, the various teaching and learning methodologies must be contrasted. This study looks at several teaching techniques that are accessible to medical students and compares them to find the ones that are most efficient and practical for both students and professors. This study attempts to determine the preferred medical education techniques among medical students as well as the caliber of the classes they attend.

## Materials and methods

Study design

This was a before-and-after study conducted among first- and second-year undergraduate students in a tertiary care teaching hospital in North India.

Study duration

The study was conducted for a period of four months, from April 2022 to July 2022. 

Size of study

The sample size was calculated using the formula z2xpx(1-p)/c, where z is the z-value, p is the percentage of picking a choice, and c is the confidence interval. Considering a 1% error and a 1.67 confidence interval, the sample variance was found to be 480. A total of 500 students, which comprised the total strength of two batches, were considered in the study.

Study population and eligibility criteria

The study population comprised first- and second-year undergraduate MBBS students of a renowned tertiary care teaching hospital situated in North India. However, those students who failed the last terminal or professional examination and those who did not give written informed consent were excluded from the study. Thus, 480 study participants who met the eligibility criteria were included in the study. 

Study procedure

Data collection began only after receiving ethical approval and administrative permission from the department's head. Following the receipt of written informed consent for participation in the study, the two batches of students were divided into three groups of 160 students each, on a random basis, using a random number generator. Each group was assigned a teacher who was responsible for teaching four short topics (decided by the common understanding and knowledge level of both the first-year and second-year students-these were topics from endocrine pharmacology, which suited the understanding level of all study participants) to the students in four different ways: traditional blackboard method, offline PowerPoint presentation, online PowerPoint presentation, and online annotative. While the students studied two distinct topics in a monitored environment using an online application and self-learning. Course materials were in line with the regulations and guidelines of the National Medical Commission’s (NMC) Undegraduate Medical Education Board’s (UGMEB) Competency-Based Medical Education (CBME) curriculum, in effect from September 2019.

To reduce bias, all three teachers would teach the three groups of students the same topic and use the same teaching method. Also, to address issues of data diffusion between students of different groups, classes of the three groups were taken by the teachers at the same date and time. All three teachers were Junior Grade Professors with minimum seven years of experience in teaching undergraduate students as full-time faculty members (contractual faculties were not included) who had an Doctor of Medicine (MD) degree in Pharmacology from an NMC (or the erstwhile Medical Council of India [MCI]) recognized medical college of India, followed by minimum three years of senior residentship in a department of Pharmacology of any medical college of the state, which included training and practice of teaching undergraduate medical students enrolled in Bachelor of Medicine and Bachelor of Surgery (in Latin: Medicinae Baccalaureus Baccalaureus Chirurgiae) (MBBS)/Bachelor of Dental Surgery (BDS) course.

Each class session lasted for 60 minutes, of which the first 10 minutes were allotted to an MCQ-based pre-test, followed by 40 minutes of teaching-learning and assessment through a post-test in the last 10 minutes, and a feedback form for that particular method was taken after the session. To assess long-term retention, a surprise post-test was conducted after 15 days. An overall feedback questionnaire for perception was administered to the students at the end of the data collection period. In the end, a teacher’s feedback form was filled out by the teachers to know their opinion as to which teaching method was preferred by them. 

Different teaching-learning methods employed

Traditional Blackboard Method

Teaching on a selected topic was conducted through a didactic method, where the teacher used the conventional blackboard and delivered the lecture.

Offline Presentation Method

The selected topic was taught using PowerPoint presentations in the classroom itself.

Online Presentation Method

The selected topic was taught using a PowerPoint presentation in online mode, where the students attended the class in a monitored environment. 

Online Annotative-Based

The selected topic was taught online by allowing students to join three different Google Meet links based on their group, and they were taught using a digital annotative device (iPad, Apple Inc., Cupertino, California, USA) and a stylus (Apple pencil, Apple Inc., Cupertino, California, USA) in a monitored environment. Any doubts thereafter were clarified on the same Google Meet platform. 

Online Application-Based

Access to the application of a certain medical coaching institution was given to the students for a certain topic. Students followed the study materials made available, which included lecture videos and ready-made notes, at their own pace and understanding throughout the class duration.

Self-Learning Method

Students were assigned a topic, and time was allotted for self-learning from any book available to them in a monitored environment. 

Data analysis

Only those students who attended all six teaching-learning classes were considered for the final analysis. The collected data was organized and tabulated in Microsoft Excel 2016 (Microsoft Office 2016 package), and statistical analysis was done using IBM Corp. Released 2015. IBM SPSS Statistics for Windows, Version 23.0. Armonk, NY: IBM Corp. The data was analyzed using appropriate statistical tools and represented by various tables, graphs, diagrams, etc. The mean and standard deviation were calculated for quantitative data. Categorical variables were expressed in proportions. For pre-post analysis, the paired-t test was used; for comparing two independent parametric variables, the unpaired t-test was used; and for comparing multiple independent parametric variables, the Analysis of Variance (ANOVA) test was used. The Dunn-Bonferroni test was used to correct other sources of error.

**Figure 1:**
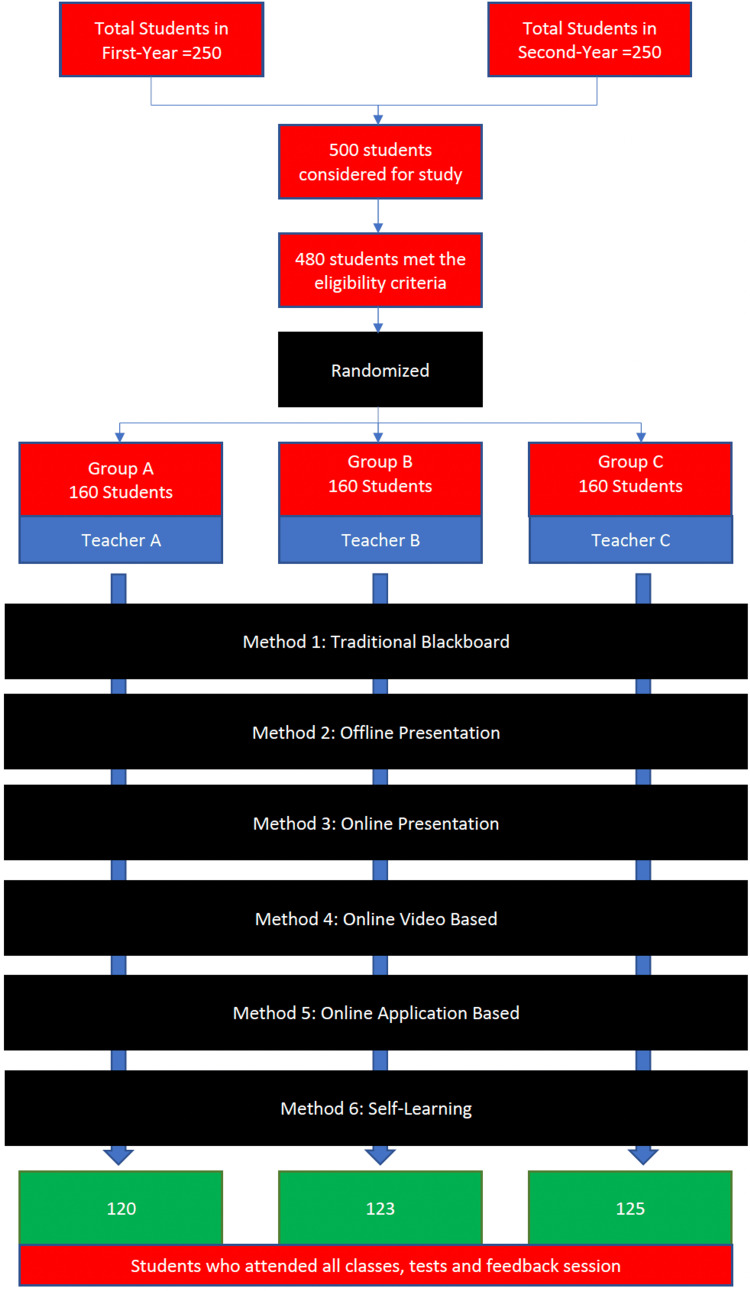
Study methodology

The entire flow of the study is depicted in Figure 1.

Ethical approval and confidentiality

Approval from the institutional ethics committee was obtained before starting the study, Reference No. 114th ECM II A/P10 (under registration number: RCR/262/INST/UP/2013/RR-19). The confidentiality of the study participants was maintained in all phases of the study. All procedures were followed according to the ethical standards of human experimentation and the Helsinki Declaration (Rev. 2013).

## Results

Out of 480 students who met the eligibility criteria, only 368 students attended all six teaching classes and completed the follow-up test after 15 days as well. Among these 368 study participants, the majority (314 students) were 18-20 years of age. Almost half (196 students) of the students were males. The proportion of students who resided in the college hostel was 320. Forty-two students came from their homes to attend classes (Table 1).

**Table 1:** Demographic profile of the study participants (N=368)

	Overall (368)		Group A (120)		Group B (123)		Group C (125)	
Demographic profile	Frequency	Percentage	Frequency	Percentage	Frequency	Percentage	Frequency	Percentage
Age								
18 to 20 years	314	85.3%	103	85.8%	109	88.6%	102	81.6%
21 to 23 years	48	13.0%	13	10.8%	13	10.6%	22	17.6%
> 23 years	6	1.6%	4	3.3%	1	0.8%	1	0.8%
Gender								
Male	196	53.3%	67	55.8%	66	53.7%	63	50.4%
Female	172	46.7%	53	44.2%	57	46.3%	62	49.6%
Residence								
Hostel	320	86.9%	104	86.7%	107	87.0%	109	87.2%
Day scholar	42	11.4%	14	11.7%	15	12.2%	13	10.4%
Rental	6	1.6%	2	1.7%	1	0.8%	3	2.4%

Table 2 compares the mean pre-test and post-test scores for the different teaching methods. For all the teaching methods except for online presentation, there was a significant improvement in the post-test scores as compared to the pre-test scores (p<0.05).

**Table 2:** Comparison of mean pre-test and post-test scores in different methods of teaching (N=368) Values are presented as n ± SD * The p-value was calculated using a paired t-test, and p<0.05 was considered to be statistically significant.

Teaching methods	Pre-test score	Post-test score	P-value
Traditional blackboard	2.86 ± 1.53	4.66 ± 2.02	0.061*
Offline presentation	3.07 ± 1.55	7.67 ± 2.65	0.001*
Online presentation	2.85 ± 1.49	3.51 ± 1.87	0.112
Online video-based	3.22 ± 1.39	5.41 ± 2.39	0.041*
Online application based	5.98 ± 2.77	8.06 ± 2.27	0.003*
Self-learning	4.41 ± 2.46	7.88 ± 2.66	0.004*

The pairwise comparison between the different teaching methods is represented in Table 3. Post-test scores of self-learning, online application-based learning, and offline presentation were remarkably better than the other methods.

**Table 3:** Comparison between post-test scores of different teaching methods (N=368) * The p-value was calculated using an unpaired t-test, and p<0.05 was considered to be statistically significant.

	Traditional blackboard	Offline presentation	Online presentation	Online video-based	Online application based	Self-learning
Traditional blackboard	--	0.002*	0.212	0.295	<0.001*	0.048*
Offline presentation	--	--	0.033*	0.047*	0.217	0.455
Online presentation	--	--	--	0.328	0.022*	0.031*
Online video-based	--	--	--	--	0.037*	0.022*
Online application based	--	--	--	--	--	0.357
Self-learning	--	--	--	--	--	--

To assess the long-term retentivity of the different teaching methods, a surprise follow-up test was conducted on the 15th day. The follow-up test scores and the post-test scores for the teaching methods were compared, and it was seen that the decline of follow-up test scores was more remarkable for traditional blackboard, offline presentation, and online video-based learning and not significant for online application-based and self-learning methods (Table 4).

**Table 4:** Comparison of mean post-test and follow-up test scores in different methods of teaching (N=368) Values are presented as n ± SD * The p-value was calculated using a paired T-test, and p<0.05 was considered to be statistically significant.

Teaching methods	Post-test score	Follow-up test score	P-value
Traditional blackboard	4.66 ± 2.02	2.74 ± 1.36	0.055*
Offline presentation	7.67 ± 2.65	2.95 ± 1.51	<0.001*
Online presentation	3.51 ± 1.87	3.53 ± 1.74	0.431
Online video-based	5.41 ± 2.39	3.68 ± 1.29	0.049*
Online application based	8.06 ± 2.27	5.91 ± 1.79	0.076
Self-learning	7.88 ± 2.66	5.98 ± 1.95	0.239

The comparison between pre-test and follow-up test scores represents whether the teaching-learning process was fruitful at all or not (Table 5). Although the post-test scores improved significantly on the same day of teaching for self-learning, online application-based learning, and offline presentation-based learning, it was noted that only scores of self-directed learning remained better in the follow-up test, implying that understanding and learning at one’s own pace had better retention than that when being taught by others. It can also be stated that if a class lecture is combined with self-learning and revision by the students, retentivity will most likely improve. Keeping this in mind, the National Medical Commission (NMC) has developed self-directed learning classes in which students analyze and revise the topics they have been taught.

**Table 5:** Comparison of mean pre-test and follow-up test scores in different methods of teaching (N=368) Values are presented as n ± SD * The p-value was calculated using a paired t-test, and p<0.05 was considered to be statistically significant.

Teaching methods	Pre-test score	Follow-up test score	p-value
Traditional blackboard	2.86 ± 1.53	2.74 ± 1.36	0.863
Offline presentation	3.07 ± 1.55	2.95 ± 1.51	0.722
Online presentation	2.85 ± 1.49	3.53 ± 1.74	0.139
Online video-based	3.22 ± 1.39	3.68 ± 1.29	0.585
Online application based	5.98 ± 2.77	5.91 ± 1.79	0.076
Self-learning	4.41 ± 2.46	5.98 ± 1.95	0.047*

Students were polled after each teaching method to determine how effective the lecture from that teaching method was for them (Table 6).

**Table 6:** Comparison of mean feedback after every teaching method

Question	Blackboard	Offline PowerPoint Presentation	Online PowerPoint Presentation	Online Annotative	Coaching App Based	Self-Study	P-value (ANOVA test)
I am confident I have learned the basic concepts taught in this class.	7.79±1.98	7.50±1.97	7.35±2.09	7.50±2.08	7.55±1.98	7.52±2.23	0.657
I believe I will be able to apply what I have learned in this class.	7.73±1.90	7.56±1.88	7.40±2.01	7.44±2.07	7.48±1.95	7.33±2.29	0.686
I am confident that I will be able to comprehend the most difficult material presented in this class’s readings.	7.55±1.95	7.24±2.04	7.23±1.95	7.34±2.04	7.33±2.00	7.39±2.19	0.662
Failure to learn the topics in this course is entirely my fault.	7.24±2.23	6.80±2.41	7.00±2.17	7.18±2.07	7.07±2.16	7.10±2.31	0.599
I was able to focus completely during the class.	7.44±1.89	7.13±1.99	7.20±2.01	7.19±2.10	7.26±2.01	7.48±2.15	0.678
The topic of this class interests me.	7.52±1.91	7.79±1.83	7.51±1.91	7.33±1.98	7.27±2.00	7.41±2.22	0.648
I believe that if I work hard enough, I will be able to grasp the class topics.	7.60±1.92	8.00±1.76	7.48±1.81	7.52±1.95	7.37±1.93	7.42±2.18	0.597
Understanding the subject matter of this class is critical to me.	7.50±2.00	7.54±2.04	7.21±2.02	7.30±1.93	7.27±1.95	7.41±2.22	0.667
I am confident that I will be able to master the skills taught in this class.	7.58±1.86	7.69±1.72	7.53±1.78	7.41±1.97	7.15±2.04	7.30±2.16	0.685
I believe I will do well in this class, given the difficulty of this course, the teacher, and my abilities.	7.63±1.89	7.73±1.74	7.45±1.86	7.43±1.92	7.21±1.96	7.40±2.21	0.624
It was easier to ask questions/doubts during this class.	7.60±1.91	7.73±1.86	7.23±2.16	7.20±2.11	7.12±2.15	7.32±2.26	0.671
The topics covered in this class were quite relevant.	7.65±1.84	7.90±1.82	7.60±1.87	7.48±2.01	7.25±2.03	7.46±2.18	0.691
During this mode of teaching, it was easier to interact with batchmates about the lecture.	7.54±1.93	7.65±1.82	7.18±2.26	7.18±2.22	7.21±2.08	7.44±2.28	0.633
I believe I will receive an excellent grade in this class.	7.66±1.99	7.51±1.70	7.40±1.90	7.39±2.03	7.31±1.99	7.43±2.24	0.617

Most students felt confident in grasping concepts and comprehending the most difficult topics taught in class when they attended class using the blackboard, and they felt the least confident when they attended class using an online PowerPoint presentation. Students believed they would be able to apply what they had learned in class and achieve excellent grades using the blackboard method, but not when doing self-study or studying using a coaching app-based method. Students believed that failure to learn the topics taught would be their fault during blackboard teaching but not when studying using an offline PowerPoint presentation. The self-study sessions allowed the students to fully concentrate in class, while the offline PowerPoint presentations caused them to lose concentration. The topic of the class was interesting for students, and they believed that if they worked hard enough, they would be able to grasp even the most difficult topics taught during the offline PowerPoint presentation, whereas they were least interested and believed the topic was difficult in the coaching app-based method. Students felt that understanding the class topic was critical and that it was easier to interact with batchmates about the lecture using the offline PowerPoint presentation method as opposed to the online PowerPoint presentation method. Students felt comfortable asking questions, thought the topics were relevant, felt confident in their ability to master the skills and perform well in the topics covered in class when presented using an offline PowerPoint presentation method, and felt least comfortable when studying using an app-based coaching method. Students believed they would be able to apply what they had learned in class and achieve excellent grades using the blackboard method, but not when doing self-study or studying using a coaching app-based method. 

Overall, student feedback indicated that they preferred the lecture via the blackboard method, followed by coaching app-based learning. The online annotative method was the least preferred (Table 7).

**Table 7:** Mean overall feedback score by students

Blackboard	Offline PowerPoint Presentation	Online PowerPoint Presentation	Online Annotative	Coaching App Based	Self-Study
7.96±2.06	6.87±2.44	6.37±2.56	6.22±2.69	7.41±2.33	7.19±2.20

According to teacher feedback, they preferred teaching via blackboard the most and least using the online annotative method (Table 8).

**Table 8:** Mean teacher’s feedback score

Blackboard	Offline PowerPoint Presentation	Online PowerPoint Presentation	Online Annotative
8.67±1.25	8.00±1.00	6.33±0.58	5.67±0.58

Table 9 compares the mean pre-test and post-test scores for each teaching method group (A, B, and C). All teaching methods showed a significant improvement in post-test scores when compared to pre-test scores. The outcome indicates that there is definitely a gain in knowledge and, thus, an improvement in scores when using any teaching method.

**Table 9:** Comparison of mean pre-test and post-test scores in different methods of teaching (N=368) * The p-value was calculated using a paired t-test, and p<0.05 was considered to be statistically significant.

	A			B			C		
	Pre-Test	Post-Test	p-Value	Pre-Test	Post-Test	p-value	Pre-Test	Post-Test	p-value
Blackboard	3.11±1.61	4.87±2.31	< 0.00001*	2.56±1.64	4.47±1.93	< 0.00001*	2.90±1.30	4.65±1.79	< 0.00001*
Offline PowerPoint Presentation	3.38±1.57	7.95±2.44	< 0.00001*	3.03±1.50	7.16±2.99	< 0.00001*	2.83±81.54	7.92±2.47	< 0.00001*
Online PowerPoint Presentation	2.87±1.70	4.29±2.01	0.000023*	2.63±1.32	3.55±1.74	0.000614*	3.05±1.43	2.65±1.48	0.000094*
Online Annotative	6.76±2.56	8.53±1.97	0.000016*	5.42±2.79	8.05±2.54	< 0.00001*	5.77±2.86	7.58±2.26	< 0.00001*
Coaching App	3.05±1.19	5.23±2.24	< 0.00001*	3.34±1.57	5.26±2.43	< 0.00001*	3.28±1.42	5.77±2.53	< 0.00001*
Self-Study	4.52±2.55	8.10±2.41	< 0.00001*	4.68±2.73	7.84±2.75	< 0.00001*	4.02±2.09	7.72±2.86	< 0.00001*

Table 10 compares the pre-test and follow-up test scores. When using the online annotative method and self-study, there was a significant improvement in follow-up test scores in group A. In group B, a significant improvement was seen after the topics were taught using an online PowerPoint presentation, an online annotative method, and during self-study, whereas no significant improvement was seen after the topics were taught using a blackboard, an offline PowerPoint presentation, or a coaching app-based method. Except for the blackboard and coaching app-based methods, all methods improved significantly in Group C.

**Table 10:** Comparison of mean pre-test and follow-up test scores in different methods of teaching (N=368) * The p-value was calculated using a paired T-test, and p<0.05 was considered to be statistically significant.

	A			B			C		
	Pre-Test	Follow-Up Test	P-value	Pre-Test	Follow-Up Test	p-value	Pre-Test	Follow-Up Test	p-value
Blackboard	3.11±1.61	2.69±1.41	0.062722	2.56±1.64	2.61±1.28	0.427489	2.90±1.30	2.92±1.41	0.418732
Offline PowerPoint Presentation	3.38±1.57	2.81±1.51	0.18363	3.03±1.50	3.16±1.61	0.323922	2.83±81.54	2.88±1.38	0.04522*
Online PowerPoint Presentation	2.87±1.70	3.31±1.68	0.076876	2.63±1.32	3.74±1.80	0.000072*	3.05±1.43	3.55±1.76	< 0.00001*
Online Annotative	6.76±2.56	2.97±1.88	< 0.00001*	5.42±2.79	2.98±1.76	< 0.00001*	5.77±2.86	2.78±1.77	< 0.00001*
Coaching App	3.05±1.19	3.10±1.40	0.418104	3.34±1.57	3.06±1.13	0.132957	3.28±1.42	3.07±1.38	0.198475
Self-Study	4.52±2.55	3.32±1.91	0.001904*	4.68±2.73	3.68±2.07	0..011676*	4.02±2.09	3.23±1.90	0 .01678*

Table 11 compares the results of the post-test and follow-up tests. In groups A and C, there was a significant improvement in follow-up test scores across all teaching methods, whereas in group B, there was a significant improvement across all teaching methods except the online PowerPoint presentation method.

**Table 11:** Comparison of mean post-test and follow-up test scores in different methods of teaching (N=368) * The p-value was calculated using a paired t-test, and p<0.05 was considered to be statistically significant.

	A			B			C		
	Post-test	Follow-up test	P-value	Post-test	Follow-up test	P-value	Post-test	Follow-up test	p-value
Blackboard	4.87±2.31	2.69±1.41	< 0.00001*	4.47±1.93	2.61±1.28	< 0.00001*	4.65±1.79	2.92±1.41	< 0.00001*
Offline PowerPoint Presentation	7.95±2.44	2.81±1.51	< 0.00001*	7.16±2.99	3.16±1.61	< 0.00001*	7.92±2.47	2.88±1.38	< 0.00001*
Online PowerPoint Presentation	4.29±2.01	3.31±1.68	0.001891*	3.55±1.74	3.74±1.80	0.27215	2.65±1.48	3.55±1.76	< 0.00001*
Online Annotative	8.53±1.97	2.97±1.88	< 0.00001*	8.05±2.54	2.98±1.76	< 0.00001*	7.58±2.26	2.78±1.77	< 0.00001*
Coaching App	5.23±2.24	3.10±1.40	< 0.00001*	5.26±2.43	3.06±1.13	< 0.00001*	5.77±2.53	3.07±1.38	< 0.00001*
Self-Study	8.10±2.41	3.32±1.91	< .00001*	7.84±2.75	3.68±2.07	< 0.00001*	7.72±2.86	3.23±1.90	< 0.00001*

## Discussion

The turbulent COVID-19 times have been devastating to the education system, and this has been particularly true for medical students. Students’ education has been greatly enhanced by online medical education in such a setting. Ever since the lockdown was put into place, experts and professionals have been developing new methods of virtual learning [6]. Many studies have been conducted on the effectiveness as well as the ease with which these methods can be used.

To balance the risk of unnecessarily exposing medical students to the virus with the need to maintain their study routines and continue their education, online medical lectures were used. Even though traditional teaching methods cannot be replaced, there is evidence in the literature to support the claim that using online teaching methods can greatly improve and modernize traditional methods [7].

The present study attempted to compare six different teaching-learning methods through teaching selected short topics to first- and second-year undergraduate medical students. In this study, the majority of the participants were male and belonged to the 18-20 years age group, which was consistent with the results of Jana et al. [5] and Joshi et al. [8].

In our study, teaching a medical topic through conventional blackboard teaching was not as effective as compared to the other methods. Until students are not actively involved through the introduction of numerous interactive approaches interspersed throughout the length of a lecture, a traditional lecture is frequently renowned for its didactic character and as the least "engaging" way of teaching. These more recent interactive approaches are more effective at achieving the intended learning outcomes, such as better problem-solving abilities and higher knowledge retention after the course [4]. The conventional method of teaching is, therefore, plagued with a higher absentee rate. In contrast, in the present study, only 28.3% of students preferred the conventional method as the mode of teaching; a study by Gupta et al. [4] reported the most preferred method of teaching to be lecturing. However, there are numerous studies that emphasize students’ preferences for interactive teaching instead of lectures, which is consistent with our study findings. Chawla et al. observed that 76% of students preferred tutorials and interactive small-group teachings, and almost all insisted that lectures should be more interactive and informative [9]. Another study by Costa et al. also showed student preference for interactive teaching as the mode of learning [10].

Teaching through offline presentation was comparatively more effective than conventional blackboard teaching and was found to be as good as other online modes of learning in the present study. This difference could be explained by the fact that the well-organized and presented content in the PowerPoint presentation was additionally explained and clarified by lectures, which supported more efficient learning. However, it is important on the part of the teacher to devise interactive content and engagingly deliver the presentation to ensure efficient and effective learning.

Online learning enables students to take part in uninterrupted online lectures given by medical educators while relaxing in their own homes, transcribe the lecture, record it, and listen to it at a time that is convenient for them and at their own speed. Students have maintained the pattern of learning during the epidemic regardless of their location by maintaining connections despite social estrangement [7]. In our study, 77.2% of the participants preferred online presentation as the mode of teaching. Online medical lectures can address most students as they encompass both auditory and visual information. It has also been established in the literature that highly motivated students are more likely to use online medical lectures [11-14]. A study done by Rafi AM et al. on conventional methods of teaching medical students based on feedback from them found that students prefer an online learning platform as a student-friendly platform where the teacher could interact with the students, and students were able to participate actively throughout the lecture [15].

Out of the other teaching-learning methods, self-learning was found to be the most effective in the study, with the most improvement in the post-test scores as compared to the pre-test. In this method, the student is at liberty to understand and study the concepts from any of the available sources or a combination of them, such as textbooks, online recorded lectures, coaching videos, and notes, as well as peer discussion. In self-directed learning, students learn at their own pace and attempt to explore various sources of information, which ensures better understanding and longer retentivity. The topics learned through the self-learning method were better retained in memory, and students performed better in the follow-up test on that topic conducted 15 days later. Studies conducted in the past have asserted that the self-directed learning method is a more effective way of delivering concepts as compared to traditional lecture sessions [16-18]. Despite better performance, only 40.8% of the students opted for the self-learning method as the preferred mode. This disparity may be due to confusion or unawareness among the students about reliable sources of information, such as reliable websites and video sources available on the internet. Nevertheless, the medical curriculum is so vast that the professors can't cover every topic during the tenure of the course. Thus, much of the learning for the students is dependent on self-directed learning. Proper guidance regarding reliable sources and scope for clarification from the professors would ensure self-learning as an essential component for gaining knowledge in the medical training course.

Limitations

First, the topics dealt with in the study were from pre-clinical subject, i.e., endocrine pharmacology, theoretical, and conducted among the first- and second-year MBBS students. Concepts that involved the demonstration of clinical skills, problem-solving, or practical exposure were not covered in the present study. Thus, other modes of teaching-learning, such as simulation-based, role play, and demonstration, were not considered in this study. Second, different modes of teaching were assessed independently. The effect of a combination of these methods or blended learning was not evaluated. Further research in this domain is recommended. Third, confounders pertaining to the studying and teaching environment weren't considered in our study.

## Conclusions

The present study highlighted the effectiveness of the different teaching-learning modes, emphasizing the shifting preferences of medical students from conventional passive learning methods to more interactive active learning methods that involved the usage of technologies. However, it is important on the part of the medical educators to acquaint themselves with the online applications. Ongoing training and updating oneself with technologies are necessary to deliver smooth and quality lectures through online platforms.

The best and most practical strategy to maintain or even raise the teaching standard is to combine the benefits of conventional and online learning to enhance medical instruction and the student experience, which is referred to as "blended teaching". Therefore, it is the responsibility of the teacher and the medical institution to prioritize the preferences of the students and facilitate the introduction of blended learning or incorporating e-learning into the educational system, even after the current pandemic situation reverses back to the earlier set-up. As opposed to lengthy classroom lectures, the use of student-friendly e-learning techniques can promote interaction between students and teachers.
